# Ostomy continence devices: a systematic review of the literature and meta‐analysis

**DOI:** 10.1111/codi.16906

**Published:** 2024-02-15

**Authors:** Justin Dourado, Zoe Garoufalia, Sameh Hany Emile, Anjelli Wignakumar, Pauline Aeschbacher, Peter Rogers, Zachary Delgado, Matthew Greer, Steven D. Wexner

**Affiliations:** ^1^ Ellen Leifer Shulman and Steven Shulman Digestive Disease Center Cleveland Clinic Florida Weston Florida USA; ^2^ Colorectal Surgery Unit, General Surgery Department Mansoura University Hospitals Mansoura Egypt; ^3^ Department of General Surgery and Bariatric and Metabolic Institute Cleveland Clinic Florida Weston Florida USA; ^4^ Department for Visceral Surgery and Medicine, Bern University Hospital University of Bern Bern Switzerland

**Keywords:** colostomy, meta‐analysis, ostomy continence devices, systematic review

## Abstract

**Aim:**

Colostomy complication rates range widely from 10% to 70%. The psychological burden on patients, leading to lifestyle changes and decreased quality of life (QoL), is one of the largest factors. The aim of this work was to assess the history and efficacy of ostomy continence devices in improving continence and QoL.

**Method:**

In this PRISMA‐compliant systematic review and meta‐analysis, we searched PubMed, Scopus, Google Scholar and clinicaltrials.gov for studies on continence devices for all ostomies up to April 2023. Primary outcomes were continence and improvement in QoL. Secondary outcomes were leakage, patient's device preference and complications. Risk of Bias 2 and the revised tool to assess risk of bias in non‐randomized studies of interventions (ROBINS‐1) were used to assess risk of bias. Certainty of evidence was graded using GRADE.

**Results:**

Twenty‐two studies assessed devices from 1978 to 2022. The two main types identified were ball‐valve devices and plug systems. Conseal and Vitala were the two main devices with significant evidence allowing for pooled analyses. Conseal, the only currently marketed device, had a pooled rate of continence of 67.4%, QoL improvement was 74.9%, patient preference over a traditional appliance was 69.1%, leakage was 10.1% and complications was 13.7%. Since 2011, five studies have investigated experimental devices on both human and animal models.

**Conclusion:**

Ostomy continence has been a long‐standing goal without a consistently reliable solution. We propose that selective and short‐term usage of continence devices may lead to improved continence and QoL in ostomy patients. Further research is needed to develop a reliable daily device for ostomy continence. Future investigation should include the needs of ileostomates.

**What does this paper add to the literature?**

Our study is the first systematic review of ostomy continence devices and includes the only quantitative pooled data comparing the outcomes of these devices. Additionally, we propose that selective usage of these devices, rather than continuous use, could lead to improved quality of life and continence in patients with ostomies.

## INTRODUCTION

An ostomy is a surgically created opening from an internal hollow organ to the outside of the body. The term ‘ostomy’ is often used interchangeably with the term ‘stoma’ [1, 2]. Ostomies are formed by exteriorizing the bowel to the abdominal wall for a variety of gastrointestinal pathologies including colorectal cancer and inflammatory bowel disease. The reported prevalence of individuals in the United States living with an ostomy ranges from 725 000 to 1 000 000. It is estimated that approximately 100 000–150 000 ostomy surgeries are performed each year [3, 4, 5]. Ostomies, while often necessary and lifesaving, are unfortunately associated with a multitude of complications, with complication rates ranging from 10% to 70% [6, 7, 8]. Common complications include peristomal skin complications, hernias, high‐output ostomies, ostomy necrosis and ostomy stenosis [9]. Additionally, one of the most prominent complications of ostomy creation is the large psychological burden that ostomies place on patients. Patients often live in fear of leakage, flatus and embarrassment from their device and its function. Because of these fears, patients often make significant changes to their daily lives, including limiting their public and social activities [2, 10].

Although ostomy‐related complications are relatively common, they can be mitigated through various strategies. One of the most common ostomy complications, skin irritation around the ostomy site [6, 11], can be addressed through a variety of products including bacterial and antifungal creams as well as skin protectants [12]. Furthermore, limiting the changing of the ostomy appliance to every 3–7 days can help prevent skin breakdown [13].

Loss of continence, or the ability to control passage of a bowel movement, places a large psychological burden on patients. Surgeons have attempted to devise surgical solutions to this problem, starting with continent ileostomy, which was first described by Kock in 1969 [14]. This procedure remains viable in select patients for whom ileoanal pouch surgery is not an option or has failed, although it has been noted to have numerous problems that necessitate repeat surgical revisions [15]. There have also been efforts to develop external devices that would allow for control of flatus and stool expulsion, referred to as ‘colostomy continence’ [16]. The first device, described in 1953, was a ‘valvular colostomy plug’ [17]. There was a gap in the literature for over 20 years before the next study on ostomy continence devices was published. The aim of the present study was to conduct a systematic review of the literature on the history, evolution and outcomes of ostomy continence devices.

## METHOD

### Registration and reporting

The protocol has been prospectively registered in the PROSPERO register of systematic reviews (CRD42023448348) and is reported according to the PRISMA 2020 guidelines [18]. There was no deviation from the registered protocol when reporting this systematic review.

### Search strategy

A systematic search of PubMed, Scopus and Google Scholar was undertaken by two investigators (ZD, ZG) for all available studies on continence devices for all ostomies up to April 2023. The investigators used the following syntax combination of keywords and Boolean operators ‘AND’ and ‘OR’ in the literature search: (device OR plug OR appliance) AND (stoma OR ostomy OR ileostomy). Additionally, clinicaltrials.gov was searched for any ongoing trials.

Conference abstracts without a full text were evaluated for their content, and if the abstract contained sufficient information for this review, the study was included. After exclusion of duplicate reports, a stepwise filtration of all remaining articles was undertaken, firstly by title and abstract followed by full‐text review of each article by two authors (SE, JD) to confirm eligibility for inclusion and extract relevant data for the systematic review and meta‐analysis. The process of literature review and article selection was under the supervision of the senior author (SDW).

### Assessment of risk of bias and certainty of evidence

Three authors (PR, PA, AW) independently assessed the risk of bias in the studies using the revised tool to assess the risk of bias in non‐randomized studies of interventions (ROBINS‐1) for cohort studies [19] and the risk of bias in randomized trials (RoB 2) for randomized trials [20]. The certainty of the evidence was graded with the GRADE approach as very low, low, moderate, or high [21]. Publication bias was assessed if 10 or more studies met the inclusion criteria.

### Data extraction and study outcomes

Three authors (PR, PA, AW) collected data including publication details (authors, year, location, journal), type of study, number of subjects and the reported outcomes, which included adjuncts used, completeness of continence, episodes of leakage, wearable time range and complications of the device, and patient perception of the device. Data collection was verified by a fourth author (JD). The primary outcomes were continence and improvement in quality of life (QoL) and the secondary outcomes were leakage rate, patient preference for the device and complications.

### Data synthesis

A meta‐analysis was conducted using the open‐source, cross‐platform software for advanced meta‐analysis, openMeta [Analyst]™ version 12.11.14. A proportional meta‐analysis was conducted to assess the weighted mean rates and 95% confidence interval (CI) of continence, patient preference, leakage, QoL and complications. Statistical heterogeneity was assessed using the inconsistency (*I* ^2^) statistic (low if *I* ^2^ < 25%, moderate if *I* ^2^ = 25%–75%, high if *I* ^2^ > 75%). A fixed‐effect meta‐analysis was used if *I* ^2^ < 25% and a random‐effect analysis was used if *I* ^2^ ≥ 25%. In case of moderate or high heterogeneity a leave‐one‐out sensitivity analysis was performed to assess for the source of heterogeneity.

## RESULTS

**FIGURE 1:**
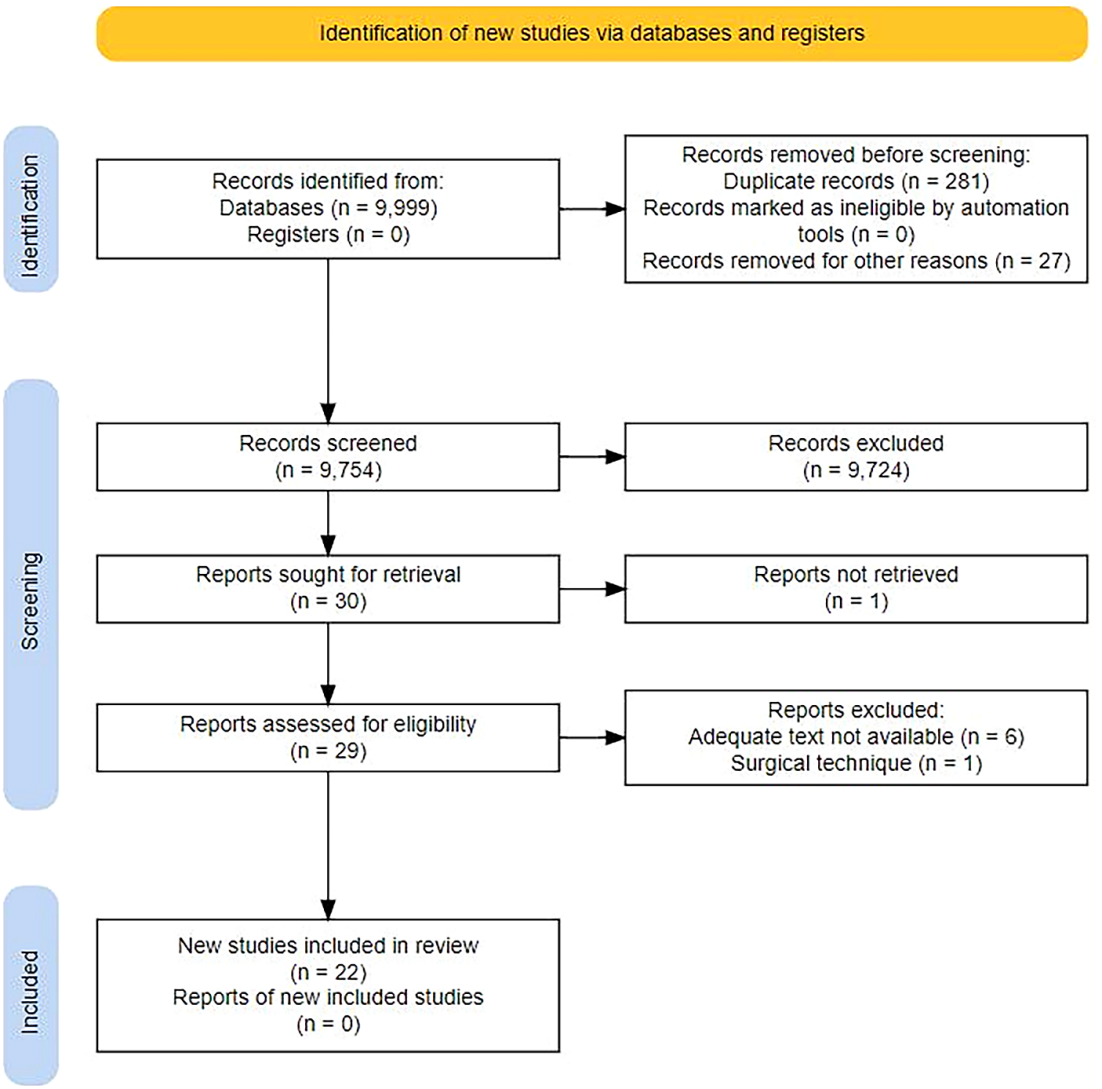
PRISMA search flowchart.

**FIGURE 2:**
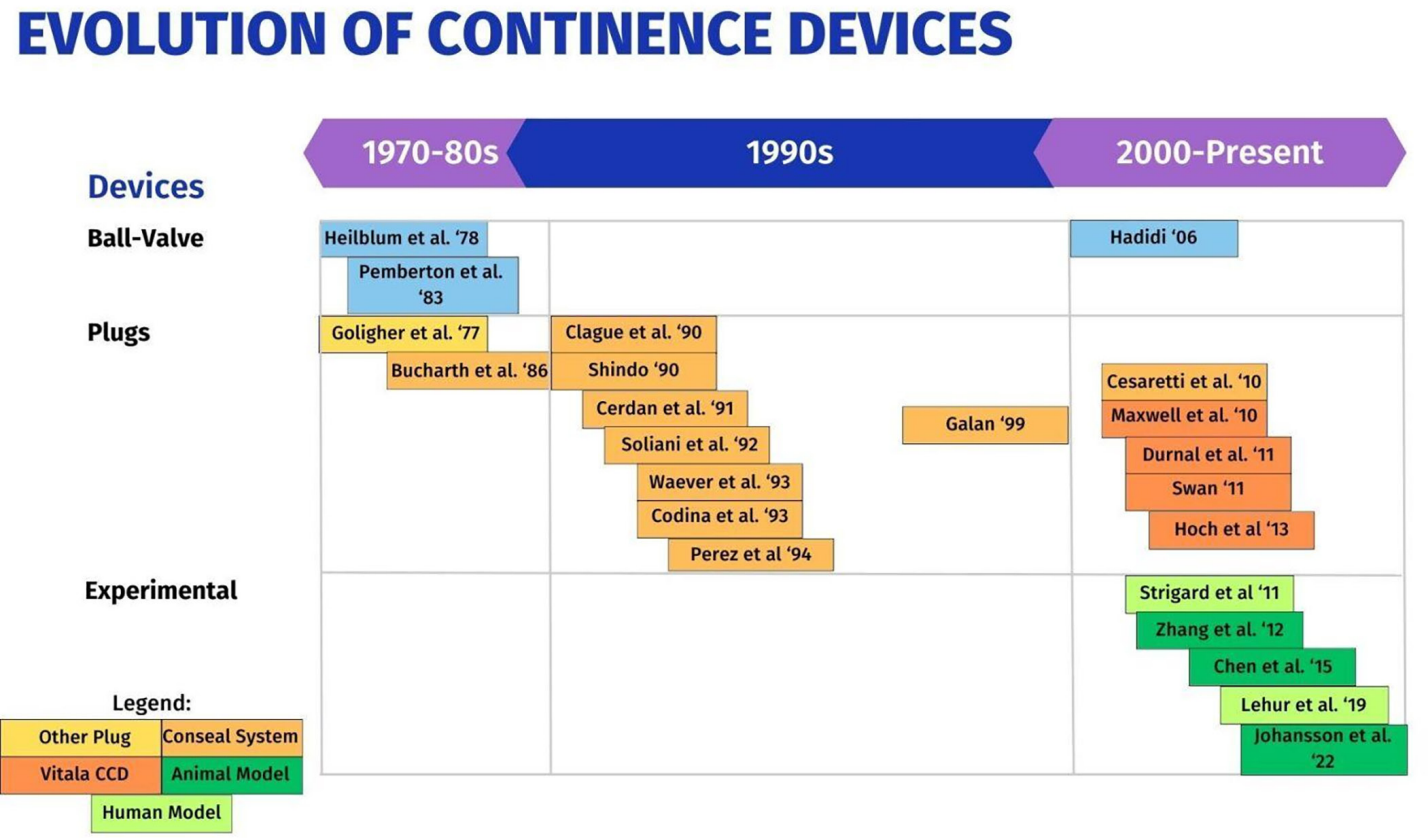
Evolution of continence devices. PRISMA 2020 flow diagram for new systematic reviews which included searches of databases and registers only.

After screening 9754 articles and abstracts, 22 articles and abstracts were ultimately included (Figure 1). There were 21 studies that were classified as a cohort or case series and one prospective randomized study. Thirteen studies were single‐centre and nine were multicentric (Table 1, Figure 2). There were no ongoing clinical trials registered. Other findings from the papers included were outside the scope of this systematic review and therefore excluded.

**TABLE 1:** Study characteristics.

Study	Journal	Year	Country	Single/multi‐centre	Study type	Device	Primary endpoint
Goliger	*Br J Surg*	1977	UK	Single	Retrospective	Erlanger magnetic ring	Continence
Heiblum (abstract)	*Dis Colon Rectum*	1978	Unknown	Single	Cohort	Ball‐valve	Continence
Pemberton	*Ann Surg*	1983	USA	Single	Prospective	Ball‐valve	Continence
Burcharth	*Lancet*	1986	Denmark	Single	Prospective	Conseal continent plug	Continence
Down	*Bristol Medico‐Chirurgical Journal*	1984	UK	Single	Prospective	Conseal continent plug	QoL
Clague (abstract)	*Surg Gynecol Obstet*	1990	UK	Multi	Prospective	Conseal continent plug	Compliance
Cerdan (abstract)	*Dis Colon Rectum*	1991	Spain	Single	Cohort	Conseal continent plug	QoL
Cesaretti	*Rev Bras Enferm*	2010	Brazil	Single	Observational	Conseal continent plug	QoL
Soliani	*Dis Colon Rectum*	1992	Italy	Single	Randomized prospective	Conseal continent plug	Patient compliance
Waever (abstract)	*Ugeskr Laeger*	1993	Denmark	Multi	Prospective	Conseal continent plug	Patient compliance
Codina	*Br J Surg*	1993	Spain	Multi	Cohort	Conseal continent plug	Patient compliance
Picon (abstract)	*Rev Esp Enferm Dig*	1994	Spain	Single	Prospective	Conseal continent plug	QoL
Hadidi	*Eur J Pediatr Surg*	2006	Germany/Egypt	Multi	Cohort	Ball‐valve	Patient compliance
Maxwell	*Dis Colon Rectum*	2010	USA	Multi	Prospective	Vitala™ CCD	QoL
Durnal	*Br J Nurs*	2011	USA	Multi	Prospective	Vitala™ CCD	QoL
Swan	*Br J Nurs*	2011	UK		Case Series	Vitala™ CCD	QoL
Hoch	*Eur J Gastroenterol Hepatol*	2013	Czech Republic	Multi	Cohort	Vitala™ CCD	QoL
Strigard	*Colorectal Dis*	2011	Sweden	Single	Prospective	Human studies	Safety and efficacy
Lehur	*Tech Coloproctol*	2019	France	Multi	Prospective	Human studies	Safety and efficacy
Zhang	*Chinese J of Tis Eng Res*	2012	China	Single	Prospective	Animal studies	Safety and efficacy
Chen	*Artif Organs*	2015	China	Single	Prospective	Animal studies	Safety and efficacy
Johansson	*J Mater Sci Mater Med*	2022	Sweden/Norway	Multi	Prospective	Animal studies	Safety and efficacy

*Abbreviations*: CCD, continence control device; QoL, quality of life.

Ostomy continence devices were broadly classified as historical devices, currently available devices and those devices under development in animal models. Overall, the articles were divided into two groups according to the type of continence device: ball‐valve devices and plug‐based devices. Within the plug devices there were two main products that were studied in the literature, the Conseal (Coloplast S.p.A., Bologna, Italy) colostomy plug system and the Vitala™ Continence Control Device (CCD) (Convatech, London, UK).

### Ball‐valve devices

The first description in the literature of a device for colostomy continence was by Wulff in 1953 [17]. Subsequently, Heilblum and Cordoba investigated an implantable silastic balloon that created a valve effect in six patients [22]. Five patients achieved continence and none of the patients experienced ischaemia of their ostomy. However, one device was removed because of infection.

The method shifted to external devices. In 1983, Pemberton et al. evaluated a balloon‐based occlusion device with a valve and found that occlusion of 5–8 h was well tolerated by four patients who achieved continence without impairing intestinal function [23]. Similarly, a study by Hadidi [24] in 2006 described a continence device that was used in 18 children ranging from 6 months to 8 years of age, with good results in continence without adverse events. Patients self‐inflated a ball with air which acted as a ball‐valve at the anorectal junction or an external appliance when used in a colostomy. This study, however, was not exclusive to ostomy patients.

### Plug devices

As with ball‐valve devices, there was an initial interest in implantable devices with plug systems. Goligher et al. described their treatment of 22 patients with an implantable magnetic ostomy device which utilized a plug system [25]. Of the 13 patients they assessed, only three (23.1%) achieved improvements in continence.

#### The Conseal device

**FIGURE 3:**
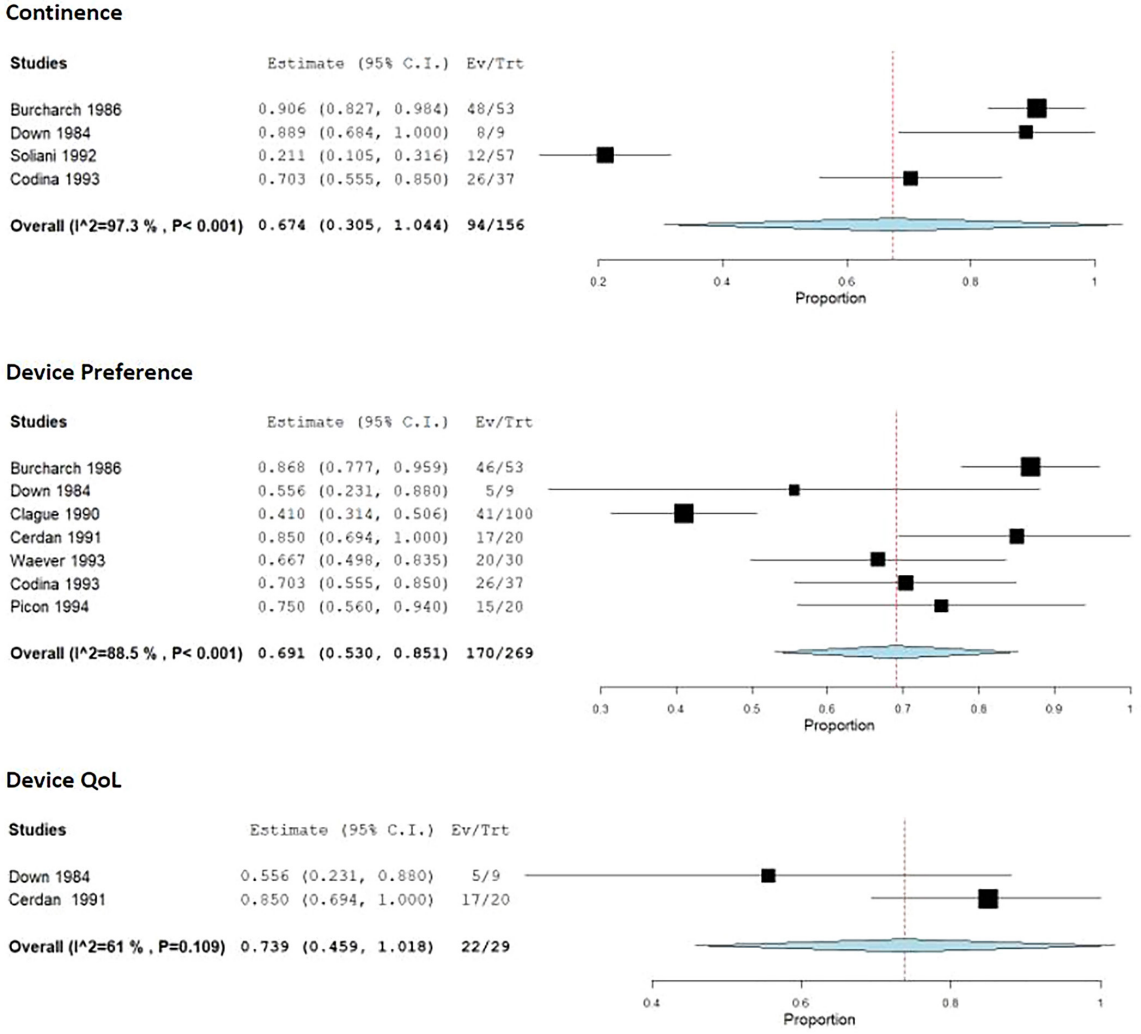
Conseal device efficacy.

The first of the widely utilized plug systems to enter the market, the Conseal, was investigated in nine studies encompassing 426 patients [26, 27, 28, 29, 30, 31, 32, 33, 34]. Proportional meta‐analysis showed that the Conseal device was associated with a 67.4% (95% CI 0.305–1.044, *I* ^2^ = 97.3%) rate of continence and 69.1% (95% CI 0.530–0.851, *I* ^2^ = 88.5%) of patients preferred the device to traditional pouching (Figure 3). Leave‐one‐out analyses of continence were performed, and when the study by Soliani et al. [31] was excluded the pooled continence rate increased from 67.4% to 83.6%. Leave‐one‐out analysis of device preference revealed no significant change in the preference rates when each study was excluded as the rates ranged from 66.3% to 76.6%.

**FIGURE 4:**
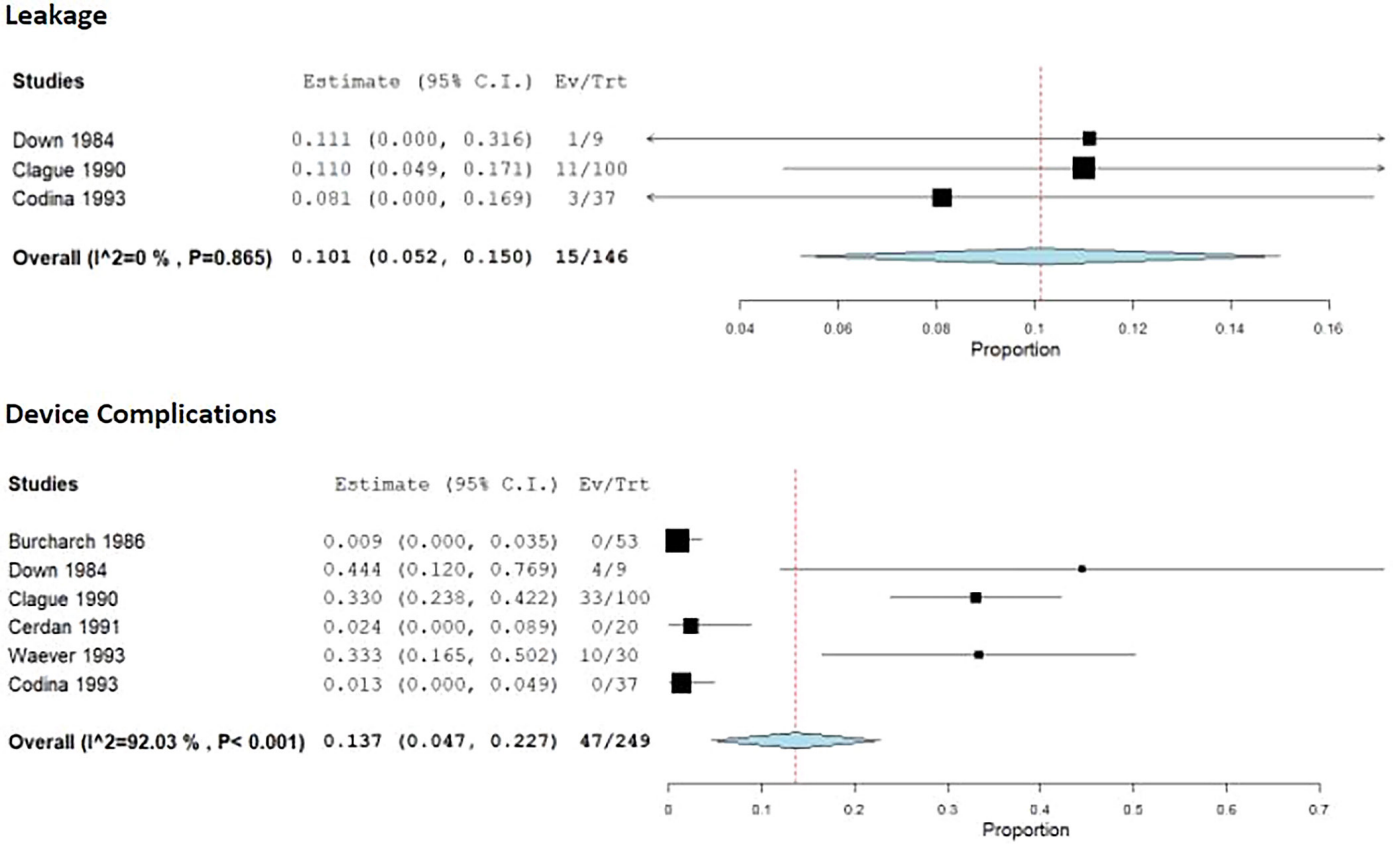
Conseal device safety.

The weighted mean rate of leakage was 10.1% (95% CI 0.052–0.150, *I* ^2^ = 0%), of QoL improvement 73.9% (95% CI 0.459–1.018, *I* ^2^ = 61%) and of complications 13.7% (95% CI 0.047–0.227, *I* ^2^ = 92.03%) (Figures 3 and 4). Leave‐one‐out analyses of QoL showed that when the study by Down and Leaper was excluded the QoL improvement increased to 85% [27]. Leave‐one‐out analyses showed high heterogeneity, in that when the studies by Burcharth et al. [26] and Codina Cazador et al. [33] were left out the complication rate increased to 19.4% and 19.3%, respectively. Conversely, when the study by Clague and Heald [28] was excluded the complication rate dropped to 6.1%. Finally, the average wear time cited in the above studies ranged from 8 to 16.5 h [26, 28, 33].

#### The Vitala™ CCD

The other device that was widely studied was the Vitala™ CCD. Four studies encompassing 271 patients were identified in the literature [16, 35, 36]. Two studies had quantitative data for analysis [16, 37]. The Vitala CCD was associated with a 69.3% (95% CI 0.605–0.782, *I* ^2^ = 0%) preference when compared with traditional ostomy appliances. Additionally, the pooled rate of leakage was 3.8% (95% CI 0.002–0.075, *I* ^2^ = 0%) and of complications was 34.1% (95% CI −0.099 to 0.780, *I* ^2^ = 96.48%). The two studies had individual complication rates of 11.5% [16] and 56.4% [37], which accounts for this high heterogeneity. Finally, the average wear time of the device in these studies ranged from 6.8 to 11 h [35, 36]. A comparison of the outcomes of the Vitala™ and Conseal devices is summarized in Table 2.

**TABLE 2:** Descriptive comparison of Conseal versus the Vitala™ CCD.

Device	Complications	Continence	Device preference	Leakage	QoL	Wear time (h)
Conseal	0.137 (0.047–0.227)	0.674 (0.305–1.044)	0.691 (0.530–0.851)	0.101 (0.052–0.150)	0.739 (0.459–1.018)	8–16.5
Vitala™ CCD	0.341 (−0.099 to 0.780)	N/a	0.693 (0.605–0.782)	0.038 (0.002–0.075)	N/a	6.8–11

*Note*: All values except for wear time presented as a pooled rate (95% confidence interval).

*Abbreviations*: CCD, continence control device; QoL, quality of life.

The effect of the Vitala CCD on improving QoL was supported by Swan [35] who combined two case studies with the above study by Durnal et al. [37] in a pooled analysis. Additionally, Hoch et al. [36] compared the Vitala CCD with traditional pouching over a 3‐month period in 165 patients. Seventy patients (42%), with a mean age of 62.5 years and with 61.2% being male, used the CCD. It was established that Vitala™ users had a higher health‐related QoL score when utilizing the EQ‐5D (*p* = 0.013) and Stoma Appliance‐specific Questionnaire (*p* = 0.001). No difference was found in the Stoma Quality of Life Scale. More Vitala users reported ‘no problems’ on all five EQ‐5D domains (*p* = 0.01), and indicated that they enjoyed life most of the time (*p* = 0.043) when compared with those having a traditional ostomy.

### Experimental devices

#### Devices under investigation in humans

There were two studies investigating new devices in human models. Strigård et al. tested a titanium transcutaneous implant evacuation system (TIES), which consisted of a titanium cylinder with a mesh and plastic cap [38], in four patients with inflammatory bowel disease who were having ostomy relocation or pouch excision. Two of the four patients needed minor corrective surgery. There were no device‐related complications or safety concerns; however, only two patients were satisfied at 18 months, with one using an ostomy bag over the TIES for extra security. Additionally, Lehur et al. tested a two‐piece appliance with a base plate and capsule cap that allowed patient‐controlled release of a folded bag in the cap to collect stool [39]. Twenty‐three patients completed the 2‐week trial: 85.3% of appliance changes were associated with no leakage whereas there were 69 incidents of leakage; 58.6% of the cap removals associated with increased pressure were manual and 41.06% were spontaneous. Adverse events were recorded in 30% of patients, some of which resolved spontaneously. However, in 13.3% of cases there was skin and subcutaneous tissue reaction which required study discontinuation. There was no difference in QoL compared with normal pouching systems and 43% said they would be willing to use it in the future.

#### Devices under development in animal models

Three studies addressed the development of additional continence devices in animal models. Zhang et al. developed an experimental model in miniature Tibetan pigs with the application of different sizes of silicone closure devices (3.0, 3.5 and 4.0 cm) [40]. They found that an occlusion diameter of 0.5–1 cm larger than the colostomy diameter has the best balance of occlusion while limiting infection, ischaemia and necrosis of the ostomy. The second study, by Chen et al., reviewed the efficacy of a pressure‐sensing colostomy plug in reduction of faecal leakage in nine miniature Tibetan pigs [41]. They were divided into 5, 10 or 15 mmHg pressure‐sensing settings. This device attempts to allow real‐time feedback to the user on the pressure within the ostomy as it relates to stool burden and the need for emptying. It was found that at 15 mmHg there was the highest sensitivity for detecting stool with no significant leakage after the first postoperative week. The final study, by Johansson et al., was specific to reservoir ileo‐ and urostomies and utilized a soft‐tissue‐anchored percutaneous port in a dog model [42]. Despite subcutaneous portion integration, the study was terminated due to adverse events related to the lack of implant–skin and implant–intestine integration. There were also issues of recurrent superficial infection surrounding the port.

### Risk of bias and certainty of evidence

Bias analyses were completed using the ROBINS‐1 and RoB 2 tools (Figures S1 and S2). Two studies had a low risk of bias, 11 studies had moderate risk of bias, eight had serious risk of bias and one did not have enough information to assess bias. Certainty of evidence was very low for all outcomes assessed (Tables S1 and S2).

## DISCUSSION

There have been no previous comprehensive reviews of ostomy continence devices. The present review found that, within the history of ostomy continence devices, two major devices were assessed in several studies that allowed pooling of their outcomes. The Conseal device, developed by Coloplast, was studied over a 24‐year period between 1986 and 2010, followed by the Vitala™ CCD, developed by ConvaTec, between 2010 and 2013. The Conseal device is the only device currently available.

Our analysis found that after the advent of ball‐valve devices, which did not have much success, the focus transitioned to plug‐based devices. When comparing the pooled outcomes of the two main plug‐based devices, the Conseal device showed an acceptably low rate of leakage and complications, with rates of continence, device preference and QoL improvement ranging from 67.4% to 73.9%. Additionally, the Conseal device had much fewer complications when compared with the Vitala CCD (13.7% vs. 34.1%). This analysis showed that, when properly used, the Conseal device could provide benefit to ostomy patients with an acceptable risk profile.

It has been shown that leakage is the criterion among ostomy patients that has the highest impact on QoL [43]. Worrying about leakage and changing lifestyle and reported wellbeing has been well documented in ostomy patients [44, 45]. Leakage is prevalent, in that the percentage of patients who report not having episodes of leakage with traditional appliances is <20% [45]. In traditional ostomy patients the Ostomy Life Study showed that 65% of ostomy patients experience leakage outside the baseplate at least once a year and 26% experience it at least monthly [46]. Additionally, they found that only 14% of patients had a leakage event less than once a year [46]. Our analysis showed that the Conseal device was able to reduce the leakage rate down to 10% while maintaining continence, which could represent an advantage.

Among patients with ostomies, a relationship has been shown between frequency of changing the appliance and increased skin complications [13]. It has also been shown that increased worry about leakage leads to more device usage [46]. These two facts, together with the fact that peristomal skin complications occur with an incidence of up to 43% [47], imply a need for a low‐leakage device that improves patients’ QoL. As previously stated, skin complications are limited with reduced leakage and when ostomy appliances are changed ideally every 3–7 days [13]. The combination of these factors makes the selection of patients who will benefit from continence devices an important consideration that is likely to have led to their lack of widespread adoption.

The Conseal device showed a good combination of improved QoL and preference over a traditional appliance with wear times of 8–16.5 h, low leakage, and low rates of complications. The authors of this study suggest that continence devices, specifically the Conseal device, could be best utilized in patients with low‐output ostomies on an as‐needed basis for life events that require increased continence and discretion to improve QoL. However, the daily usage of this device is not recommended after our review, as frequent changes could increase skin complications.

Five experimental devices have been investigated since 2011 in both human and animal models. Both human models utilized an implantable baseplate and a plug system with two animal models using a plug system and one ball‐valve system. None of these experimental devices reached optimal satisfaction or complication rates. This result further emphasizes that ostomy continence is relevant to patient care and QoL but a durable and reliable solution is not available.

The limitations of our study are the high heterogeneity, low certainty of evidence and the moderate to serious risk of bias in the included studies. As only one study was a randomized trial, the retrospective and descriptive nature of the studies included, in addition to the large time span over which they were performed, probably accounts for these limitations. To minimize the heterogeneity, leave‐one‐out analyses were performed. The increased continence after the exclusion of the study by Soliani [31] is probably because this study reported continence over a 48‐h period rather than per device usage as reported in the other studies. This led the authors to conclude that the possible increase in continence is more pronounced with shorter wear and selective rather than continuous usage. The increased complications after the exclusion of the studies by Burcharth et al. [26] and Codina Cazador et al. [33] is probably because these studies reported no complications.

## CONCLUSION

Ostomy continence has been a long‐standing goal without a reliable daily solution. We propose that the selective and short‐term usage of continence devices could potentially lead to improved continence and QoL in patients with ostomies. Further research is needed to develop a reliable daily device for ostomy continence. In addition, continence for ileostomates also requires investigation.

## AUTHOR CONTRIBUTIONS

**Steven D. Wexner:** Conceptualization; project administration; supervision. **Justin Dourado:** Conceptualization; investigation; writing – original draft; methodology; validation; visualization; formal analysis; data curation. **Zoe Garoufalia:** Investigation; data curation; formal analysis; writing – review and editing. **Sameh Hany Emile:** Investigation; writing – review and editing; formal analysis; data curation. **Anjelli Wignakumar:** Investigation; writing – review and editing; formal analysis; data curation. **Pauline Aeschbacher:** Investigation; writing – review and editing; formal analysis; data curation. **Peter Rogers:** Investigation; writing – review and editing; formal analysis; data curation. **Zachary Delgado:** Investigation; writing – review and editing; formal analysis; data curation. **Matthew Greer:** Investigation; writing – review and editing; formal analysis; data curation.

## FUNDING INFORMATION

None.

## CONFLICT OF INTEREST STATEMENT

Dr Wexner is a consultant for ARC/Corvus, Baxter, Becton, Dickinson and Co, GI Supply, Glaxo Smith Kline, ICON Clinical Research Ltd, Intuitive Surgical, Leading Biosciences/PalisadeBio, Livsmed, Medtronic, Olympus, OstomyCure, Stryker, Takeda, Virtual Ports, is a member of the Data Safety Monitoring Board of JSR/WCG/ACI (chair), Polypoid (chair) and Boomerang, and receives royalties from Intuitive Surgical Karl Storz Endoscopy America Inc., Medtronic and Unique Surgical Solutions, LLC.

## ETHICS STATEMENT

No ethic committee approval was required for this kind of studies (Literature review).

## Supporting information


Figure S1:

Figure S2:

Table S1:

Table S2:

## Data Availability

By reasonable request to first author.
