# Supplementary figures and images for: Ostomy continence devices: a systematic review of the literature and meta‐analysis
Source: Colorectal Dis. 2024 Feb 15;26(4):622–31. doi: 10.1111/codi.16906 (PMC12150823; doi:10.1111/codi.16906)

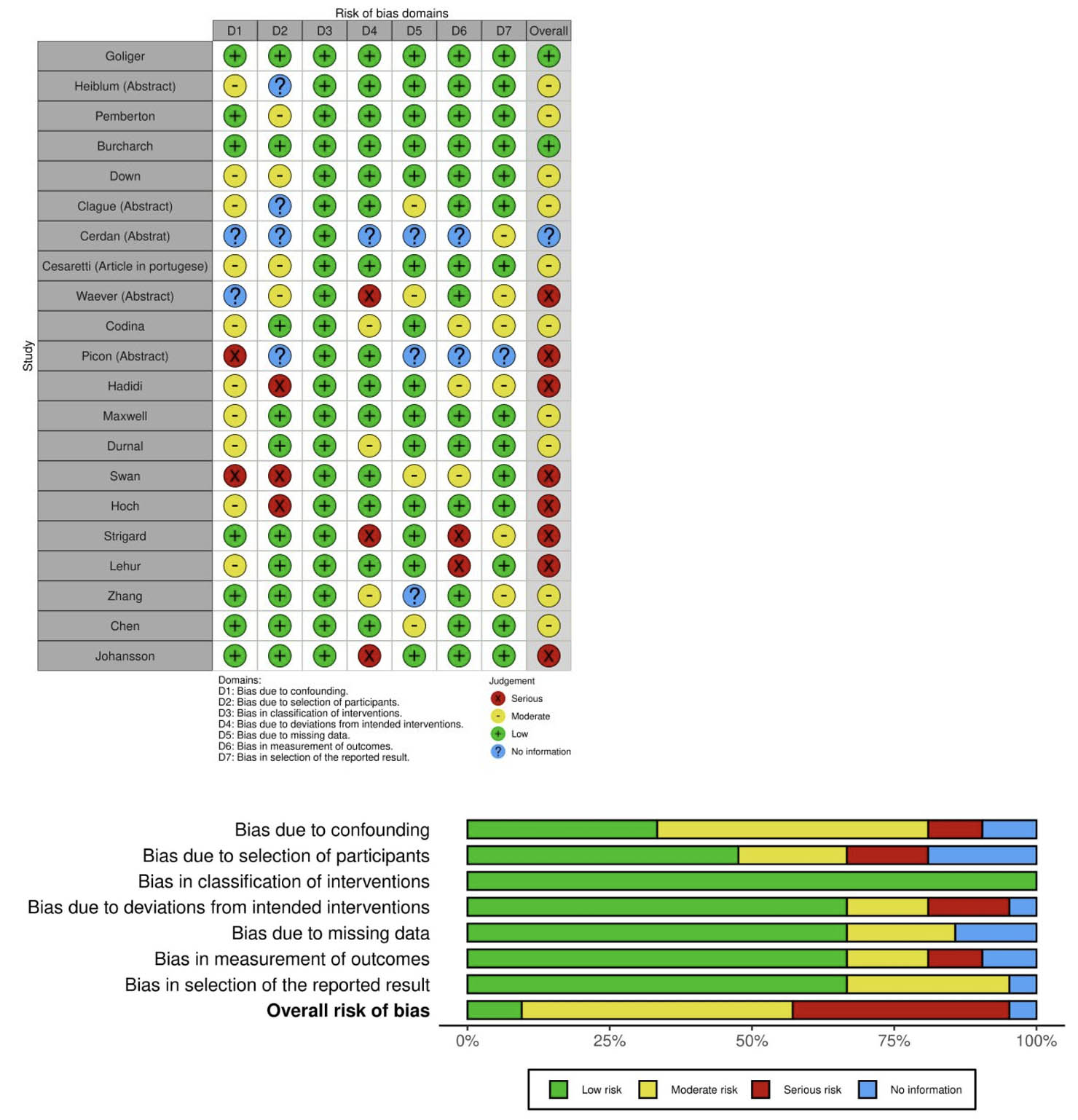

Supplement: Supplementary file 1 — Figure S1: [file CODI-26-622-s001.jpg]

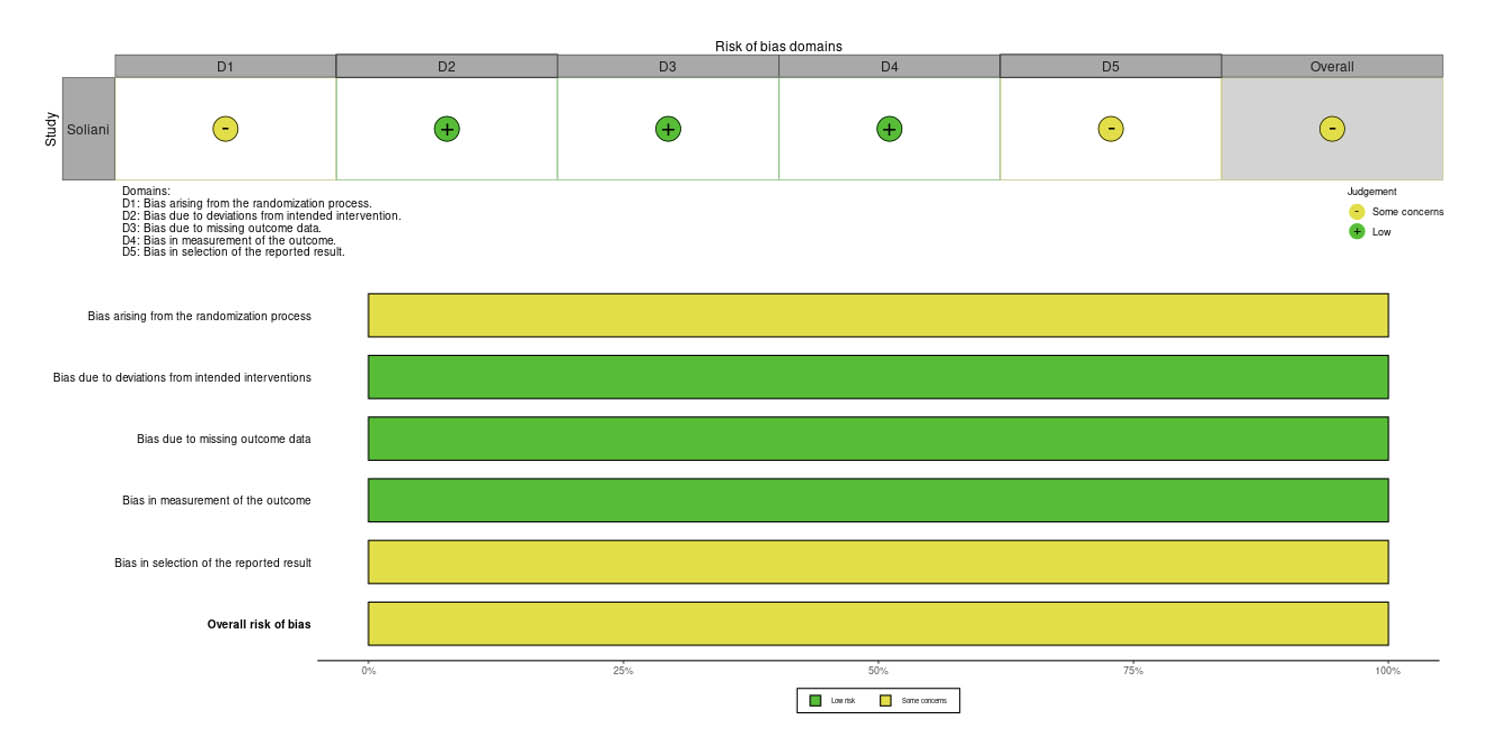

Supplement: Supplementary file 2 — Figure S2: [file CODI-26-622-s004.jpg]
