# Supplementary material for: Ostomy continence devices: a systematic review of the literature and meta‐analysis
Source: Colorectal Dis. 2024 Feb 15;26(4):622–31. doi: 10.1111/codi.16906 (PMC12150823; doi:10.1111/codi.16906)
Supplement: Supplementary file 3 — Table S1: [file CODI-26-622-s002.docx]

**Supplementary Table 1: GRADE Analysis of Certainty of Evidence (Conseal Device)**

| **Certainty assessment** | | | | | | | **№ of patients** | **Effect** | **Certainty** | **Importance** |
| --- | --- | --- | --- | --- | --- | --- | --- | --- | --- | --- |
| **№ of studies** | **Study design** | **Risk of bias** | **Inconsistency** | **Indirectness** | **Imprecision** | **Other considerations** | **the Conseal devices** | **Relative** **(95% CI)** |  |  |
| **Continence** | | | | | | | | | | |
| 4 | Randomised trial and observational studies | Serious^a^ | Very serious^b^ | Not serious | Not serious | None | 94/156 | **Rate ratio 0.647** **(0.305 to 1.044)** | ⨁◯◯◯ Very low | CRITICAL |
| **Device Preference** | | | | | | | | | | |
| 7 | Observational studies | Serious^a^ | Very serious^b^ | Not serious | Not serious | None | 170/269 | **Rate ratio 0.691** **(0.530 to 0.851)** | ⨁◯◯◯ Very low | CRITICAL |
| **Leakage** | | | | | | | | | | |
| 3 | Observational studies | Serious^a^ | Not serious | Not serious | Not serious | None | 15/146 | **Rate ratio 0.101** **(0.052 to 0.150)** | ⨁◯◯◯ Very low | IMPORTANT |
| **QoL** | | | | | | | | | | |
| 2 | Observational studies | Serious^a^ | Serious^b^ | Not serious | Not serious | None | 22/29 | **Rate ratio 0.739** **(0.459 to 1.018)** | ⨁◯◯◯ Very low | IMPORTANT |
| **Complications** | | | | | | | | | | |
| 6 | Observational studies | Serious^a^ | Very serious^b^ | Not serious | Not serious | None | 47/249 | **Rate ratio 0.137** **(0.047 to 0.227)** | ⨁◯◯◯ Very low | IMPORTANT |

**CI:** confidence interval

#### Explanations

a. Moderate to Serious risk of bias in multiple studies

b. High heterogeneity (I^2)
