# Supplementary material for: Ostomy continence devices: a systematic review of the literature and meta‐analysis
Source: Colorectal Dis. 2024 Feb 15;26(4):622–31. doi: 10.1111/codi.16906 (PMC12150823; doi:10.1111/codi.16906)
Supplement: Supplementary file 4 — Table S2: [file CODI-26-622-s003.docx]

**Supplementary Table 2: GRADE Analysis of Certainty of Evidence (Vitala CCD)**

| **Certainty assessment** | | | | | | | **№ of patients** | **Effect** | **Certainty** | **Importance** |
| --- | --- | --- | --- | --- | --- | --- | --- | --- | --- | --- |
| **№ of studies** | **Study design** | **Risk of bias** | **Inconsistency** | **Indirectness** | **Imprecision** | **Other considerations** | **the Vitala CCD** | **Relative** **(95% CI)** |  |  |
| **Device preference** | | | | | | | | | | |
| 2 | Observational studies | Serious^a^ | Not serious | Not serious | Not serious | None | 72/104 | **Rate ratio 0.693** **(0.605 to 0.782)** | ⨁◯◯◯ Very low | CRITICAL |
| **Leakage** | | | | | | | | | | |
| 2 | Observational studies | Serious^a^ | Not serious | Not serious | Not serious | None | 4/104 | **Rate ratio 0.038** **(0.002 to 0.075)** | ⨁◯◯◯ Very low | IMPORTANT |
| **Complications** | | | | | | | | | | |
| 2 | Observational studies | Serious^a^ | Very serious^b^ | Not serious | Not serious | None | 47/104 | **Rate ratio 0.341** **(-0.099 to 0.780)** | ⨁◯◯◯ Very low | IMPORTANT |

**CI:** confidence interval

#### Explanations

a. Moderate to Serious risk of bias in multiple studies

b. High heterogeneity (I^2)
